# Interleukin 4–driven loss of stromal LIF signaling affects immune responses and cell-cell adhesion in atopic dermatitis

**DOI:** 10.1016/j.isci.2026.116766

**Published:** 2026-07-13

**Authors:** Yarden Feller, Kiril Malovitski, Sari Assaf, Yan Stein, Sivan Friedman, Dafna Tussia-Cohen, Lubna Khair, Rawaa Ishtewy, Tzachi Hagai, Avraham Unterman, Ofer Sarig, Eli Sprecher, Liat Samuelov

**Affiliations:** 1 Division of Dermatology, Tel Aviv Sourasky University Medical Center, Tel Aviv, Israel; 2 The Gray Faculty of Medical & Health Sciences, Tel Aviv University, Tel Aviv, Israel; 3 The Genomic Research Laboratory for Lung Fibrosis, Tel Aviv Sourasky University Medical Center, Tel Aviv, Israel; 4 The Evolutionary Genomics of Host-Pathogen Interactions Laboratory, Faculty of Life Sciences, Tel Aviv University, Tel Aviv, Israel

**Keywords:** atopic dermatitis, LIF, interleukin-6, DSG1, cell-cell adhesion

## Abstract

Atopic dermatitis (AD) involves immune dysregulation, epidermal barrier defects, and impaired cell-cell adhesion, yet the contribution of dermal fibroblasts (FBs) remains poorly understood. Here we show that leukemia inhibitory factor (LIF), a cytokine produced by dermal FBs, is downregulated in AD skin—driven by the Th2 cytokine IL-4—and that this loss triggers a self-amplifying inflammatory circuit. Using single-cell RNA sequencing, immunofluorescence, keratinocyte (KCs)-FBs co-cultures, and functional adhesion assays, we demonstrate that reduced LIF signaling elevates IL-6 production in KCs, activates ERK1/2, and decreases membrane expression of the desmosomal protein desmoglein 1 (DSG1), impairing epidermal cohesion. DSG1 loss further amplifies IL-6 secretion, perpetuating the cycle. Blocking IL-6 or ERK1/2 signaling rescues adhesion defects. These findings identify a stromal-epidermal axis linking Th2 inflammation to barrier dysfunction and offer mechanistic insights into AD chronicity.

## Highlights

• IL-4 downregulates LIF expression in dermal fibroblasts of patients with atopic dermatitis
• Loss of fibroblast-derived LIF impairs keratinocyte cell-cell adhesion via IL-6 and ERK1/2
• DSG1 downregulation amplifies IL-6 secretion, creating a self-reinforcing inflammatory loop
• Blocking IL-6 or ERK1/2 signaling rescues LIF knockdown-induced adhesion defects

## Graphical abstract

**Figure:**
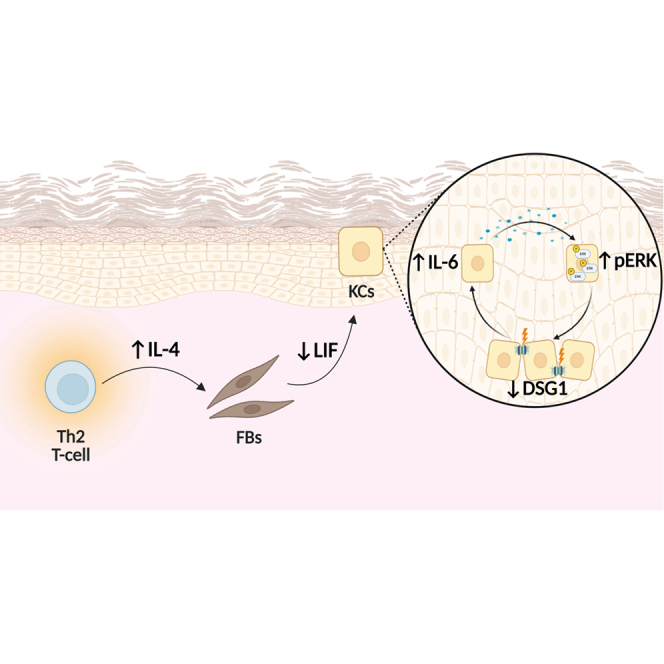

## Teaser

Biological sciences

## Introduction

Atopic dermatitis (AD) is a common chronic inflammatory skin disorder characterized by eczematous lesions, intense pruritus, and a relapsing course.^1^ Immune dysregulation with Th2 skewing and epidermal barrier disruption play a role in the pathogenesis of AD and contribute to chronic inflammation and disease manifestations.^2^^,^^3^^,^^4^

Over the past years, the study of adhesion molecules in monogenic disorders associated with an atopic-like phenotype has suggested a critical role for cell-cell adhesion in AD pathogenesis.^5^^,^^6^ Adhesion molecules are not only essential for preserving keratinocytes (KCs) cohesion; they play a pivotal role in the maintenance of the skin barrier function^7^^,^^8^^,^^9^ and the regulation of inflammatory circuits.^10^ Molecules associated with those rare single-gene disorders, such as desmoglein 1 (DSG1), are abnormally expressed or dysfunctional in AD skin.^6^^,^^11^ Of interest, cytokines upregulated in AD have been shown to downregulate the expression of critical adhesion proteins,^12^ underscoring the interdependency of epidermal and immune elements in AD pathogenesis.

DSG1 is a major adhesion protein responsible for the integrity of cell-cell adhesion in the upper epidermal layers. In addition to its role in epidermal adhesion, DSG1 also regulates KCs differentiation through the modulation of the ERK/Erbin pathway.^13^^,^^14^ The pathophysiology of AD has been linked to defective DSG1 expression, which compromises barrier function and thereby increases vulnerability to allergens and environmental irritants.^5^^,^^15^^,^^16^ Unlike pemphigus foliaceus or pemphigus vulgaris, where autoantibodies directly disrupt DSG1 and DSG3, causing acantholysis and blistering, DSG1 down-regulation in AD results from cytokine- and protease-driven mechanisms. This results in partial adhesion loss and barrier fragility rather than frank blistering.

Although epidermal and immune dysregulation plays a major role in AD,^17^ the molecular pathways and specific cell populations mediating crosstalk between dermal elements, including dermal fibroblasts (FBs), and the immune system or the epidermis in AD are yet to be defined.^18^^,^^19^^,^^20^^,^^21^^,^^22^ Dermal-epidermal crosstalk and continuous communication between FBs and KCs is crucial for maintaining skin homeostasis and promoting critical physiological processes such as cell division/proliferation and wound healing.^18^^,^^23^^,^^24^ Leukemia inhibitory factor (LIF), a member of the interleukin (IL)-6 family of cytokines that signals through the Janus kinase-STAT3 pathway, is expressed by both dermal FBs and epidermal KCs^25^ and serves as one of the molecules that mediate this dermal-epidermal crosstalk.^19^^,^^20^ LIF is known to play a role in regulating inflammation and controlling epidermal proliferation and differentiation.^25^^,^^26^^,^^27^^,^^28^^,^^29^^,^^30^ In addition, reduced LIF expression has been identified in AD FBs, suggesting a direct role of LIF in AD pathogenesis and impaired epidermal barrier function.^19^^,^^20^

Here we show that reduced FBs-derived LIF contributes to AD pathogenesis by triggering a self-amplifying mechanism involving IL-6 and DSG1. Rather than initiating AD, this pathway likely amplifies barrier dysfunction and inflammation, possibly underlying AD chronicity.

## Results

### LIF expression is reduced in AD patient-derived dermal FBs

**Figure 1:**
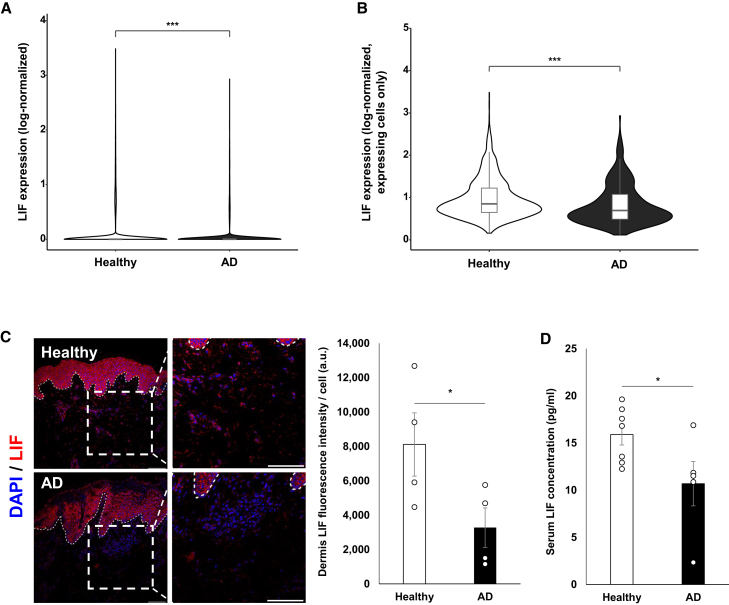
LIF expression is reduced in AD patient-derived dermal fibroblasts (A) Violin plot shows *LIF* expression in dermal fibroblasts (FBs) from patients with AD and healthy controls, based on single-cell RNA sequencing of lesional skin from 6 patients with AD and 6 healthy controls. Each violin represents the distribution of log-normalized expression across all FBs cells per group. Statistical significance was determined by a Wilcoxon rank-sum test (log2FC = −0.79, padj = 3.2 × 10^−15^). (B) Violin plot shows *LIF* expression among *LIF*-expressing dermal FBs from patients with AD and healthy controls. Statistical significance was determined by Wilcoxon rank-sum test restricted to LIF-expressing cells (log2FC = −0.30, padj = 2.1 × 10^−15^). (C) Immunofluorescence staining of LIF (red) and DAPI (blue) in healthy and AD skin samples (dashed line marks the dermal-epidermal junction, scale bars, 100 μm). Quantification represents mean fluorescence intensity normalized to the number of DAPI^+^ cells in the dermis. Individual data points represent values from each patient. Data are shown as mean ± SEM; *n* = 4 patients per group; *t* test. (D) Serum LIF concentrations measured by ELISA in patients with AD (*n* = 5) and healthy controls (*n* = 7). Individual data points represent values from each subject. Data are shown as mean ± SEM; *t* test. a.u. = arbitrary units. Asterisks denote: ∗*p* < 0.05.

To achieve cell-type-specific resolution of LIF expression in the dermis, single-cell RNA sequencing (scRNAseq) was performed on lesional skin from 6 patients with AD and 6 healthy controls (Table S1). Per-donor Uniform Manifold Approximation and Projection (UMAP) projections were generated to assess individual-level consistency (Figure S1A). PERMANOVA (999 permutations, Euclidean distance) confirmed that disease status was the primary source of compositional variance, with no significant contribution from gender, age, or sampling location (Table S2). Following quality control, the single-cell data included 108,145 high-quality cells. Using a standard Seurat scRNA-seq analysis pipeline,^31^^,^^32^^,^^33^^,^^34^ based on previously reported markers,^35^^,^^36^^,^^37^^,^^38^^,^^39^^,^^40^^,^^41^ we identified 28 distinct cell subsets (Figures S2A and S2B). Of these, approximately 38,400 were identified as dermal FBs. Single-cell differential expression analysis revealed significantly reduced LIF expression in AD FBs compared to healthy controls (log2FC = −0.79, padj = 3.2 × 10^−15^, Figure 1A). As cytokines are known to display inherently high cell-to-cell transcriptional variability in single-cell data, being expressed in only a subset of cells at any given time,^42^ we further examined LIF expression within LIF-expressing FBs specifically. This analysis confirmed significantly reduced LIF expression among AD LIF-expressing FBs (log2FC = −0.30, padj = 2.1 × 10^−15^, Figure 1B), indicating that the overall reduction reflects both a decrease in the proportion of LIF-expressing cells (7.0% in AD compared to 9.8% in controls) and reduced expression per expressing cell. This reduction was observed across multiple FBs subpopulations (Figure S2C) and was consistent across individual donors (Figure S2D).

To validate the findings identified using scRNAseq, we performed immunofluorescence (IF) studies on skin biopsies obtained from involved skin of patients with AD and healthy controls (Table S1). In line with the scRNAseq findings, these studies showed lower LIF expression in AD skin, with a 60% decrease in dermal LIF expression compared to healthy controls (Figure 1C), which is in line with previously published data.^19^^,^^20^ To assess whether LIF downregulation extends systemically, we measured serum LIF and found significantly lower LIF concentrations in patients with AD compared with healthy controls (Figure 1D). These data raised the possibility that an element associated with AD pathogenesis may be responsible for inhibiting LIF expression in FBs.

### IL-4 negatively regulates LIF expression in FBs

**Figure 2:**
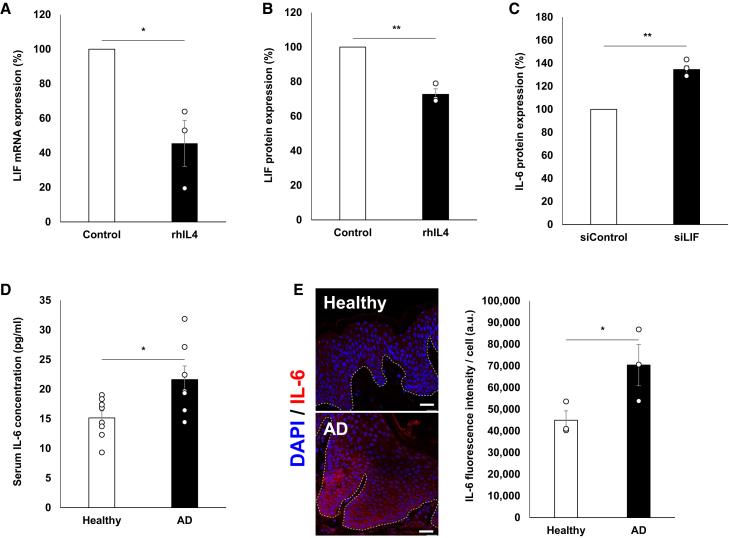
Consequences of LIF downregulation in dermal fibroblasts and keratinocytes (A) Fibroblasts (FBs) were supplemented with 20 ng/mL rhIL-4 and cultured for 72 h. Total RNA was extracted, and *LIF* mRNA levels were quantified by qRT-PCR, normalized to *ACTB*. Individual data points represent values from each independent experiment. Data are shown as mean ± SEM of three independent experiments, expressed as percentage relative to control; paired *t* test. (B) FBs were supplemented with 20 ng/mL rhIL-4 and cultured for 72 h. LIF protein levels in the supernatants were quantified by ELISA. Individual data points represent values from each independent experiment. Data are shown as mean ± SEM of three independent experiments, expressed as percentage relative to control; paired *t* test. (C) Keratinocytes were transfected with siLIF or siControl. IL-6 protein levels in the supernatants were quantified by ELISA. Individual data points represent values from each independent experiment. Data are shown as mean ± SEM of three independent experiments, expressed as percentage relative to siControl; paired *t* test. (D) Serum IL-6 concentrations measured by ELISA in healthy controls (*n* = 8) and patients with AD (*n* = 7). Individual data points represent values from each subject. Data are shown as mean ± SEM; *t* test. (E) Immunofluorescence staining of IL-6 (red) and DAPI (blue) in healthy and atopic skin sections (dashed line marks the dermal-epidermal junction, scale bars, 100 μm). Quantification represents mean fluorescence intensity normalized to the number of DAPI^+^ cells in the epidermis. Individual data points represent values from each patient. Data are shown as mean ± SEM; *n* = 3 patients per group; *t* test. a.u. = arbitrary units. Asterisks denote: ∗*p* < 0.05, ∗∗*p* < 0.001.

IL-4 is a central Th2 cytokine implicated in AD pathogenesis.^43^ To test whether IL-4 contributes to the reduction of LIF in dermal FBs, primary human FBs were treated with recombinant human (rh) IL-4. IL-4 exposure significantly downregulated LIF at both the mRNA and protein levels (Figures 2A and 2B; Figures S3A–S3D). These findings are consistent with prior reports of IL-4-mediated LIF down-regulation in other tissues,^44^^,^^45^ and may account for the reduced LIF expression observed in AD FBs.

To further investigate the downstream effects of reduced LIF signaling on KCs, and to enable molecular analyses, *LIF* knockdown (KD) was achieved in primary human KCs using *LIF*-specific small interfering RNA (siRNA). Enzyme-linked immunosorbent assay (ELISA) analysis of supernatants revealed significantly increased IL-6 secretion (Figure 2C). Accordingly, serum IL-6 concentrations were significantly higher in patients with AD compared with healthy controls (Figure 2D), although within the normal range.^46^ In addition, IL-6 immunoreactivity was upregulated in involved skin of patients with AD compared to healthy controls (Figure 2E). Since IL-6 signaling is known to increase ERK1/2 phosphorylation,^47^^,^^48^ we investigated pERK levels following *LIF* KD in primary KCs. In line with the elevated IL-6 levels, LIF downregulation in KCs resulted in increased levels of pERK (Figure S3E).

### Decreased LIF expression impairs epidermal cell-cell adhesion

**Figure 3:**
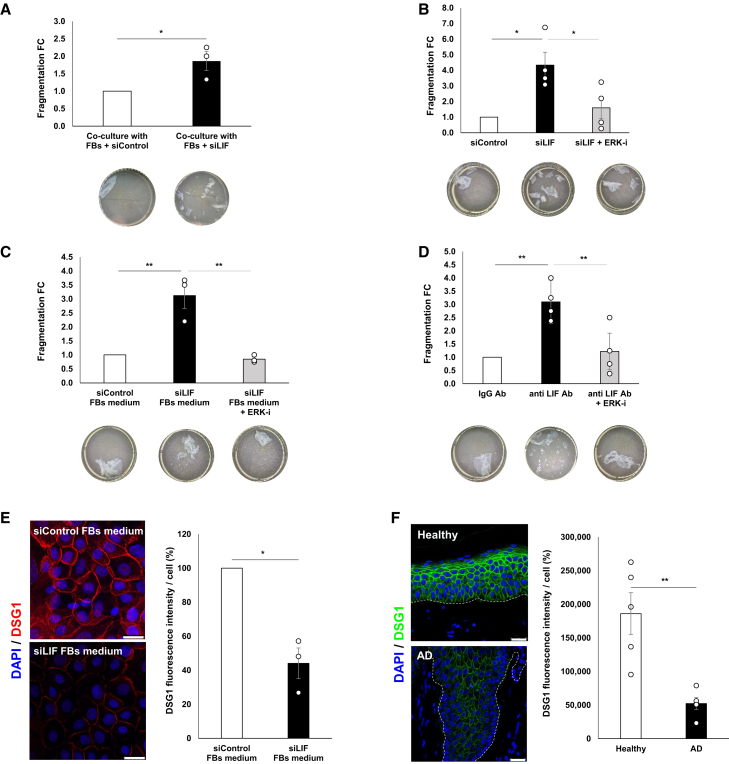
LIF deficiency impairs epidermal cell-cell adhesion and DSG1 expression (A) Keratinocytes (KCs) were co-cultured with fibroblasts (FBs) treated with siLIF or siControl. KCs adhesion was assessed by a dispase dissociation assay. Individual data points represent values from each independent experiment. Data are shown as mean ± SEM of three independent experiments, expressed as fold change (FC) of KCs fragments relative to siControl; paired *t* test. (B) KCs were transfected with siLIF or siControl and cultured with or without ERK inhibitor (ERK-i). KCs adhesion was assessed by a dispase dissociation assay. Individual data points represent values from each independent experiment. Data are shown as mean ± SEM of four independent experiments, expressed as FC relative to siControl; one-way ANOVA with post-hoc Tukey HSD test. (C) Conditioned medium from siLIF- or siControl-treated FBs was applied to KCs cultured with or without ERK-i. KCs adhesion was assessed by a dispase dissociation assay. Individual data points represent values from each independent experiment. Data are shown as mean ± SEM of three independent experiments, expressed as FC relative to siControl; one-way ANOVA with post-hoc Tukey HSD test. (D) KCs were cultured with a LIF neutralizing antibody or normal IgG control, with or without ERK-i. KCs adhesion was assessed by a dispase dissociation assay. Individual data points represent values from each independent experiment. Data are shown as mean ± SEM of four independent experiments, expressed as FC relative to IgG; one-way ANOVA with post-hoc Tukey HSD test. (E) Conditioned medium from siLIF- or siControl-treated FBs was applied to KCs, then subjected to immunofluorescence (IF) staining for DSG1 (red) and DAPI (blue) (scale bars, 25 μm). Quantification represents mean fluorescence intensity normalized to the number of DAPI^+^ cells. Individual data points represent values from each independent experiment. Data are shown as mean ± SEM of three independent experiments, expressed as percentage relative to siControl; *t* test. (F) IF staining of DSG1 (green) and DAPI (blue) in healthy and AD skin samples (dashed line marks the dermal-epidermal junction, scale bars, 25 μm). Quantification represents mean fluorescence intensity normalized to the number of DAPI^+^ cells in the epidermis. Individual data points represent values from each patient. Data are shown as mean ± SEM; *n* = 5 patients per group; *t* test. a.u. = arbitrary units. Asterisks denote: ∗*p* < 0.05, ∗∗*p* < 0.001.

Elevated ERK1/2 activity is known to impair desmosomal adhesion,^49^ and defective intercellular adhesion contributes to AD pathogenesis.^5^^,^^6^^,^^7^^,^^50^ To test whether reduced FBs-derived LIF affects KCs adhesion, we first used a co-culture system in which KCs were cultured with *LIF*-KD FBs (Figure S4A). Dispase dissociation assays revealed increased fragmentation of KCs monolayers (Figure 3A), establishing that loss of FBs-derived LIF is sufficient to impair epidermal adhesion. To dissect the underlying mechanism, we employed complementary approaches. Direct LIF KD in KCs similarly resulted in increased fragmentation, which was reversed by ERK inhibition (Figure 3B). Consistent with these findings, KCs cultured with conditioned medium from LIF-KD FBs (Figure S4B) or with a LIF neutralizing antibody also demonstrated increased fragmentation, which was normalized following ERK inhibition (Figures 3C and 3D), suggesting that reduced LIF signaling impairs epidermal intercellular adhesion through ERK1/2 activation.

To elucidate the mechanisms by which LIF affects cell-cell adhesion in the epidermis, we investigated the expression of adhesion molecules following *LIF*-KD. IF and immunoblotting studies demonstrated reduced DSG1 expression in KCs cultured with medium obtained from *LIF*-KD FBs compared to control FBs (Figure 3E) and in *LIF*-KD KCs (Figure S5A), respectively. To further support these findings, we showed that DSG1 immunoreactivity was downregulated in involved skin of patients with AD compared to healthy controls (Figure 3F). In contrast, the expression of the desmosomal cadherin desmocollin 1 (DSC1), remained unchanged following *LIF*-KD in KCs (Figures S5B and S5C). These findings suggested that reduced LIF expression, as demonstrated in AD-involved skin, results in impaired epidermal adhesion through reduced DSG1 expression.

### DSG1 downregulation in keratinocytes upregulates IL-6 expression

**Figure 4:**
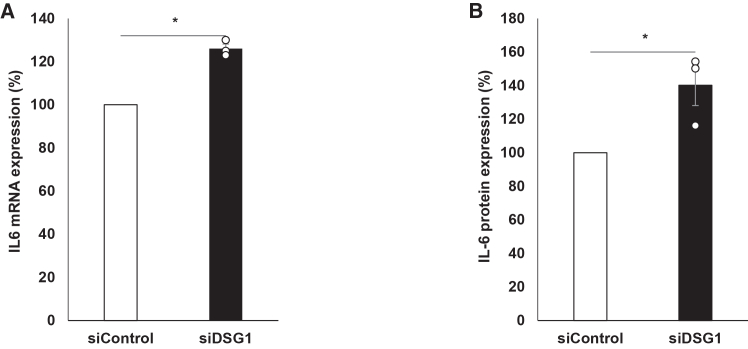
DSG1 knockdown upregulates IL-6 expression in keratinocytes (A) Keratinocytes (KCs) were transfected with siDSG1 or siControl. Total RNA was extracted, and *IL6* mRNA levels were quantified by qRT-PCR, normalized to *ACTB*. Individual data points represent values from each independent experiment. Data are shown as mean ± SEM of three independent experiments, expressed as percentage relative to siControl; *t* test. (B) KCs were transfected with siDSG1 or siControl. IL-6 protein levels were quantified from cell lysates by ELISA. Individual data points represent values from each independent experiment. Data are shown as mean ± SEM of three independent experiments, expressed as percentage relative to siControl; *t* test. Asterisks denote: ∗*p* < 0.05.

Besides its role in epidermal adhesion, DSG1 is involved in various cellular processes, including signal transduction, cell proliferation, and differentiation.^13^^,^^16^^,^^51^ Here we aimed at investigating whether reduced DSG1 expression in KCs as a result of LIF downregulation has a role in mediating inflammatory responses. *DSG1* was knocked down in KCs using *DSG1*-specific siRNA, and cytokine expression levels were assessed. In line with previous studies,^16^^,^^52^^,^^53^^,^^54^ *DSG1* KD resulted in significantly upregulated IL-6 levels both at the RNA and protein levels, using real-time quantitative reverse transcription PCR (qRT-PCR) and ELISA, respectively (Figures 4A and 4B).

### Blocking IL-6 signaling rescues *LIF* knockdown-associated impaired adhesion

**Figure 5:**
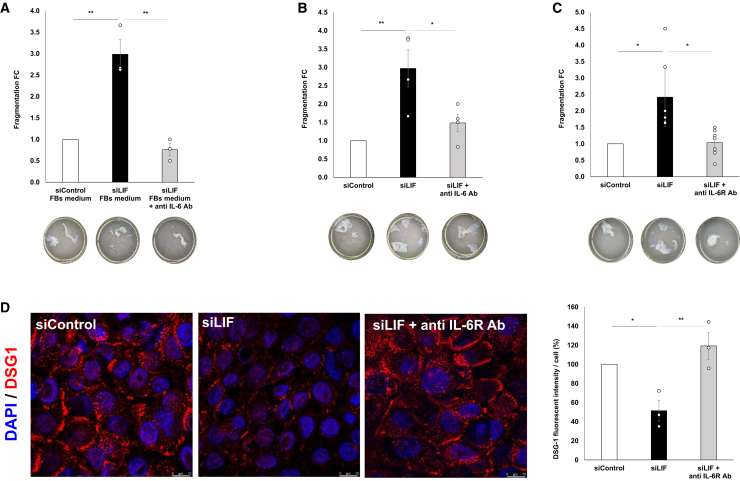
IL-6 blocking rescues adhesion defect in LIF-deficient keratinocytes (A) Conditioned medium from siLIF- or siControl-treated fibroblasts was applied to keratinocytes (KCs) with or without neutralizing anti-IL-6 antibody (anti-IL-6 Ab). KCs adhesion was assessed by a dispase dissociation assay. Individual data points represent values from each independent experiment. Data are shown as mean ± SEM of three independent experiments, expressed as fold chane (FC) relative to siControl; one-way ANOVA with post-hoc Tukey HSD test. (B) KCs were transfected with siLIF or siControl and cultured with or without anti-IL-6 Ab. KCs adhesion was assessed by a dispase dissociation assay. Individual data points represent values from each independent experiment. Data are shown as mean ± SEM of four independent experiments, expressed as FC relative to siControl; one-way ANOVA with post-hoc Tukey HSD test. (C) KCs were transfected with siLIF or siControl and cultured with or without IL-6 receptor-blocking antibody (anti-IL-6R Ab). KCs adhesion was assessed by a dispase dissociation assay. Individual data points represent values from each independent experiment. Data are shown as mean ± SEM of seven independent experiments, expressed as FC relative to siControl; one-way ANOVA with post-hoc Tukey HSD test. (D) KCs were transfected with siLIF or siControl and cultured with or without anti-IL-6R Ab, then subjected to immunofluorescence staining for DSG1 (red) and DAPI (blue) (scale bars, 10 μm). Quantification represents mean fluorescence intensity normalized to the number of DAPI^+^ cells. Individual data points represent values from each independent experiment. Data are shown as mean ± SEM of three independent experiments, expressed as percentage relative to siControl; one-way ANOVA with post-hoc Tukey HSD test. Asterisks denote: ∗*p* < 0.05 and ∗∗*p* < 0.001.

Taken collectively, the results presented above suggest the existence of a self-amplifying pathophysiological circuit involving LIF, IL-6, and DSG1 which may, at least in part, contribute to the chronic inflammatory state typical of AD. To determine whether interrupting this loop could restore KCs adhesion, we first tested the effects of IL-6 neutralization. KCs cultured either with conditioned medium from *LIF*-KD FBs (Figure S4B) or following direct *LIF* KD, exhibited increased fragmentation in the dispase dissociation assay. Treatment with a neutralizing anti-IL-6 antibody (anti-IL-6 Ab) corrected this phenotype (Figures 5A and 5B).

*LIF*-KD KCs were then treated with an IL-6 receptor (IL-6R)—blocking antibody (anti-IL-6R Ab), which selectively inhibits IL-6 signaling without affecting receptors for other IL-6 family cytokines, including the LIF receptor and gp130.^55^ IL-6R blockade, which has demonstrated clinical effectiveness in several inflammatory disorders,^56^ including AD,^57^ significantly rescued the adhesion impairment induced by LIF downregulation, as measured by the dispase dissociation assay (Figure 5C). Moreover, IL-6R blocking reversed the reduction in DSG1 expression caused by *LIF* KD in IF studies (Figure 5D).

These findings suggest that IL-6 signaling plays a critical role in reduced LIF-induced adhesion defects, and that blocking IL-6 signaling can potentially restore DSG1 expression and impaired adhesion.

## Discussion

Our study identifies reduced FBs-derived LIF as a mediator of epidermal dysfunction in AD. Our observations suggest a model according to which IL-4, a pivotal Th2 cytokine known to drive AD pathogenesis, downregulates LIF expression in FBs; loss of LIF signaling results in increased IL-6 production by KCs, subsequent ERK1/2 activation, and downregulation of DSG1, ultimately impairing epidermal cell-cell adhesion; DSG1 deficiency in turn leads to increased IL-6 production, suggesting a self-reinforcing inflammatory circuit underlying AD pathogenesis. These findings reveal a mechanistic link between dermal FBs and epidermal barrier defects in AD. Importantly, key findings—including impaired adhesion upon loss of LIF signaling and rescue by ERK1/2 inhibition or IL-6 signaling blockade—were reproduced across three complementary experimental systems, supporting the robustness of the proposed pathway.

The IL-4-LIF-IL-6-DSG1 axis uncovered here provides an explanation for how Th2 cytokines may indirectly disrupt epidermal adhesion. Most current models of AD pathogenesis emphasize immune dysregulation, filaggrin deficiency, and intrinsic KCs abnormalities. However, emerging single-cell and spatial transcriptomic studies have suggested a more active role for mesenchymal cells in disease progression. Our results extend these observations by showing that FBs-derived LIF not only supports epidermal homeostasis but also actively restrains inflammatory signaling through the suppression of IL-6. The identification of a stromal-epidermal communication pathway that regulates desmosomal integrity adds an additional layer to the pathophysiology of AD. IL-6 signaling emerges as a mechanistic contributor to adhesion defects; however, current IL-6 inhibitors have shown limited efficacy in AD.^57^ Our findings therefore highlight IL-6 as a pathway of interest rather than an established therapeutic target.

LIF is a pleiotropic cytokine that belongs to the IL-6 family. Besides the skin, LIF is expressed in a wide range of tissues, including the nervous system, bone, and hematopoietic cells, where it plays a role in neuronal survival, bone formation, and hematopoiesis.^58^^,^^59^ In the skin, LIF regulates cell growth and differentiation as well as inflammation^25^^,^^26^^,^^27^^,^^28^^,^^29^^,^^30^ in part through regulating the secretion of several cytokines in a range of cell types.^26^^,^^45^^,^^60^ LIF exerts its effects by binding to a receptor complex composed of LIF receptor and gp130. This receptor complex is shared with other cytokines including ciliary neurotrophic factor and oncostatin M, indicating overlapping signaling.^58^^,^^59^ These support the role of LIF in mediating inflammation in AD^25^^,^^30^^,^^61^; however other studies suggest that LIF is primarily an anti-inflammatory cytokine that may oppose inflammation in certain contexts.^62^^,^^63^ This indicates that LIF’s function can vary significantly based on disease nature and cellular environment, highlighting the complexity of its mechanisms of action.

DSG1 is a major component of desmosomes, which is crucial for epidermal cell adhesion in the upper epidermal layers; however, it also plays a significant role in regulating cytokine expression and inflammation. DSG1 deficiency leads to a Th17-skewed inflammatory response in both mice and humans.^9^ It also suppresses epidermal growth factor receptor (EGFR) signaling which is essential for normal KCs differentiation and homeostasis,^64^^,^^65^ maintains epidermal barrier integrity and regulates gap junction turnover, both of which are essential for controlling inflammation.^51^^,^^66^ Overall, DSG1 influences inflammation through cytokine regulation, EGFR signaling modulation, and barrier function maintenance.

In conclusion, this study provides insights into how low levels of FBs-derived LIF contribute to the pathogenesis of AD by weakening adhesion and fueling inflammation, contributing to disease persistence and flares. The identification of a pathophysiological network involving IL-4, IL-6, DSG1, and LIF offers promising avenues for therapeutic development in AD.

### Limitations of the study

Several limitations of this study merit consideration. The transcriptomic findings are based on a modest cohort size, which is inherent to studies involving primary human skin biopsies, and replication in larger and more diverse patient cohorts would further strengthen the conclusions. The *in vitro* mechanistic experiments relied on primary KCs and FBs derived from foreskin or plastic surgery donors rather than lesional AD skin, which may not fully recapitulate the complexity of the *in vivo* inflammatory microenvironment. Although three complementary experimental systems were employed, direct extraction of RNA and protein from KCs in the co-culture transwell model was not technically feasible, necessitating the use of conditioned media and direct knockdown approaches for molecular analyses. The serum LIF measurements were performed in a limited number of subjects and should be regarded as exploratory. Finally, while IL-6 signaling blockade rescued adhesion defects in our experimental model, the modest magnitude of IL-6 induction and the limited clinical efficacy of IL-6 inhibitors reported in AD suggest that the identified pathway likely acts in concert with other disease mechanisms rather than as a standalone driver of AD pathogenesis.

## Resource availability

### Lead contact

Requests for further information and resources should be directed to and will be fulfilled by the lead contact, Eli Sprecher (elisp@tlvmc.gov.il).

### Materials availability

This study did not generate new unique reagents.

### Data and code availability

• Single-cell RNA sequencing data generated in this study have been deposited in NCBI’s Gene Expression Omnibus and are publicly available as of the date of publication (GEO: GSE288946).
• This paper does not report original code. All scRNA-seq analyses were performed using publicly available software as described in the method details section.
• Any additional information required to reanalyze the data reported in this paper is available from the lead contact upon request.

## Acknowledgments

We would like to express our gratitude to our patients and their families for participating in the present study. The study was supported in part by a grant award from the Israel Science Foundation (grant no. 2792/20) and by a grant from the Ichilov-Safra TAU initiative in computational biology.

## Author contributions

Y.F.: conceptualization, formal analysis, investigation, validation, and writing—original draft; K.M.: investigation and validation; S.A.: investigation and validation; Y.S.: methodology and formal analysis; S.F.: investigation and formal analysis; D.T.C.: investigation and formal analysis; L.K.; investigation and validation; R.I.: investigation and validation; T.H.: methodology and formal analysis; A.U.: methodology and formal analysis; O.S.: formal analysis, investigation, methodology, and project administration; E.S.: conceptualization, funding acquisition, resources, supervision, and writing—review and editing; L.S.: conceptualization, funding acquisition, supervision, and writing—review and editing.

## Declaration of interests

The authors declare no competing interests.

## Declaration of generative AI and AI-assisted technologies in the writing process

During the preparation of this work, the authors used Claude (Anthropic) in order to assist with manuscript editing and formatting. After using this tool, the authors reviewed and edited the content as needed and take full responsibility for the content of the publication.

## STAR★Methods

### Key resources table

**Table:** 

REAGENT or RESOURCE	SOURCE	IDENTIFIER
**Antibodies**		

Anti-LIF	Abcam, UK	Cat# ab138002; RRID: AB_3083551
Anti-IL-6	R&D Systems, USA	Cat# AF-206-NA; RRID: AB_354392
Anti-DSG1	Santa Cruz Biotechnology, USA	Cat# sc-137164; RRID: AB_2093310
Anti-α-Tubulin	Abcam, UK	Cat# ab15246; RRID: AB_301787
Anti-DSC1	Santa Cruz Biotechnology, USA	Cat# sc-398590; RRID: AB_2894905
Anti-ERK1/2	Santa Cruz Biotechnology, USA	Cat# sc-514302; RRID: AB_2571739
Anti-pERK	Santa Cruz Biotechnology, USA	Cat# sc-16982-R; RRID: AB_653182
HRP-conjugated goat anti-mouse secondary	Jackson ImmunoResearch Laboratories, USA	Cat# 115-035-003; RRID: AB_10015289
HRP-conjugated goat anti-rabbit secondary	Sigma-Aldrich, USA	Cat# 12-348; RRID: AB_390191
Alexa Fluor 568-conjugated goat anti-rat	Abcam, UK	Cat# ab175476; RRID: AB_2813739
Alexa Fluor 488-conjugated donkey anti-mouse	Jackson ImmunoResearch Laboratories, USA	Cat# 715-545-150; RRID: AB_2340846
Alexa Fluor 647-conjugated goat anti-mouse	Abcam, UK	Cat# ab150115; RRID: AB_2687948
Alexa Fluor 568-conjugated donkey anti-goat	Invitrogen, USA	Cat# A11057; RRID: AB_142581
Human LIF neutralizing	R&D Systems, USA	Cat# AB-250-NA; RRID: AB_354316
Anti-IL-6 neutralizing	Abcam, UK	Cat# ab6672; RRID: AB_2127460
IL-6R blocking	Roche, Germany	RRID: AB_3694993
Normal IgG control	R&D Systems, USA	Cat# AB-108-C; RRID: AB_354267

**Biological samples**		

Lesional skin biopsies from AD patients (scRNAseq; *n* = 6)	Tel Aviv Sourasky University Medical Center IRB	N/A
Healthy skin biopsies, controls (scRNAseq; *n* = 6)	Tel Aviv Sourasky University Medical Center IRB	N/A
Lesional skin biopsies from AD patients (IF; *n* = 6)	Tel Aviv Sourasky University Medical Center IRB	N/A
Healthy skin biopsies, controls (IF; *n* = 6)	Tel Aviv Sourasky University Medical Center IRB	N/A
Serum samples, AD patients	Tel Aviv Sourasky University Medical Center IRB	N/A
Serum samples, healthy controls	Tel Aviv Sourasky University Medical Center IRB	N/A
Primary human keratinocytes	Tel Aviv Sourasky University Medical Center IRB	N/A
Primary human dermal fibroblasts	Tel Aviv Sourasky University Medical Center IRB	N/A

**Chemicals, peptides, and recombinant proteins**		

Recombinant human IL-4 (rhIL-4)	R&D Systems, USA	Cat# 204-IL
SCH772984 (ERK1/2 inhibitor)	Selleckchem, USA	Cat# S7101
Dispase II	Roche, Germany	Cat# 04942078001
Lipofectamine RNAiMAX transfection reagent	Invitrogen, USA	Cat# 13778150
CelLytic M cell lysis reagent	Sigma-Aldrich, USA	Cat# C2978
Protease inhibitor cocktail	Sigma-Aldrich, USA	Cat# P8340
Clarity Western ECL substrate	Bio-Rad, USA	Cat# 1705061
Bovine serum albumin (BSA)	Sigma-Aldrich, USA	Cat# A7906
Citrate buffer for antigen retrieval	Invitrogen, USA	Cat# 005000

**Critical commercial assays**		

LIF ELISA kit	Abcam, UK	Cat# ab242228
IL-6 ELISA kit	R&D Systems, USA	Cat# D6050B
RNA extraction kit	Roche, Germany	Cat# 11828665001
qScript cDNA synthesis kit	Quanta Biosciences, USA	Cat# 95047-100
PerfeCTa SYBR Green FastMix	Quanta Biosciences, USA	Cat# 95072-012
KC growth medium	Lonza, USA	Cat# 00192060
Human Whole Skin Dissociation Kit	Miltenyi Biotec, USA	Cat# 130-101-540
Chromium Next GEM Single Cell 3′ Kit v3.1	10× Genomics, USA	Cat# PN-1000269

**Deposited data**		

scRNAseq raw and processed data	This paper; NCBI GEO	GEO: GSE288946

**Experimental models: Cell lines**		

Primary human keratinocytes	This paper	N/A
Primary human dermal fibroblasts	This paper	N/A

**Oligonucleotides**		

siRNA targeting LIF	Santa Cruz Biotechnology, USA	Cat# sc-37222
siRNA targeting DSG1	Santa Cruz Biotechnology, USA	Cat# sc-35224
Stealth RNAi siRNA Negative Control Lo GC	Invitrogen, USA	Cat# 12935200
qRT-PCR primer DSG1 Fwd: CCCTCCAGTGTTTTCAATGGC	This paper	N/A
qRT-PCR primer DSG1 Rev: AATTGTTCGGTTCATCTGCG	This paper	N/A
qRT-PCR primer LIF Fwd: ATACGCCACCCATGTCAC	This paper	N/A
qRT-PCR primer LIF Rev: CCACATAGCTTGTCCAGGTTG	This paper	N/A
qRT-PCR primer ACTB Fwd: ACCTTCTACAATGAGCTGCG	This paper	N/A
qRT-PCR primer ACTB Rev: CCTGGATAGCAACGTACATGG	This paper	N/A
qRT-PCR primer GAPDH Fwd: GAGTCAACGGATTTGGTCGT	This paper	N/A
qRT-PCR primer GAPDH Rev: GACAAGCTTCCCGTTCTCAGCC	This paper	N/A
qRT-PCR primer IL6 Fwd: GTAGTGAGGAACAAGCCAGAGC	This paper	N/A
qRT-PCR primer IL6 Rev: TACATTTGCCGAAGAGCCCT	This paper	N/A

**Software and algorithms**		

Cell Ranger v6.0	10× Genomics	https://support.10xgenomics.com
Seurat v4.1.1	Hao et al.^31^	https://satijalab.org/seurat/
Imaris 10	BitPlane, USA	https://imaris.oxinst.com/
ImageJ/Fiji	Schindelin et al.^67^; NIH	https://imagej.net/software/fiji/
R (UMAP/DimPlot)	R Core Team	https://www.r-project.org/

**Other**		

6-well transwell inserts	Greiner Bio-One, Austria	Cat# 657641
Chromium Next GEM Chip G	10× Genomics, USA	Cat# PN-1000127
NovaSeq 6000 sequencing platform	Illumina, USA	N/A
SP8 inverted confocal microscope	Leica Microsystems, Germany	N/A
Leica HC PL APO CS2 x63/1.4 NA objective	Leica Microsystems, Germany	N/A
Leica HC PL APO CS2 x20/0.75 NA objective	Leica Microsystems, Germany	N/A
StepOnePlus qPCR system	Applied Biosystems, USA	N/A
Gradient gel for electrophoresis	Bio-Rad, USA	Cat# 5678093
Nitrocellulose membrane	Bio-Rad, USA	Cat# 1704159
Cellometer ViaStain AOPI Staining Solution	Revvity, USA	Cat# CS2-0106-5 ML
Chromium Controller platform	10× Genomics, USA	N/A
gentleMACS Dissociator	Miltenyi Biotec, USA	Cat# 130-093-235

### Experimental model and study participant details

#### Human subjects and skin samples

Skin samples were obtained from 6 naive AD patients and 11 healthy controls following written informed consent in accordance with a protocol approved by the Tel Aviv Sourasky University Medical Center Institutional Review Board (IRB; ethics approval number: 0966-20-TLV) and in adherence with the principles of the Declaration of Helsinki. AD patient samples were used for both scRNAseq and immunofluorescence analyses. For healthy controls, 6 donors were used for scRNAseq, and 6 donors were used for immunofluorescence (including 1 donor shared between both analyses). Detailed demographic information for all donors including age, sex, disease severity scores (IGA, SCORAD, NRS), and sampling location is provided in Table S1. The influence of sex on the results was examined as part of the inter-individual variability analysis (Table S2); no significant effect of sex on cell-type composition was detected. Ancestry, race, and ethnicity were not systematically recorded for this cohort.

#### Primary cell cultures

Human primary keratinocytes (KCs) and dermal fibroblasts (FBs) were isolated from healthy donors’ skin - male (foreskin) or female (obtained from plastic surgeries). All primary cell cultures were routinely tested for mycoplasma contamination and confirmed negative. Primary cells from multiple independent donors were used across experiments; the number of biological replicates per experiment is indicated in the corresponding figure legends.

### Method details

#### Human subjects and skin samples

To evaluate potential sources of variability introduced by donor demographics and sampling site, we performed principal component analysis (PCA) on cell-type composition profiles across all scRNAseq 12 donors (Figures S1B–S1D). PERMANOVA (999 permutations, Euclidean distance) was used to formally quantify the contribution of disease status, gender, age, and sampling location to compositional variance (Table S2).

#### Primary cell cultures

Primary KCs were maintained in KC growth medium (Lonza, USA). FBs were cultured in Dulbecco’s Modified Eagle Medium (DMEM) supplemented with 10% fetal bovine serum (Biological Industries, Israel). All cells were maintained at 37°C in a humidified atmosphere with 5% CO_2_. To investigate dermal-epidermal crosstalk, co-cultures were established using 6-well inserts (657641, Greiner bio-one, Austria), with FBs seeded on plates and KCs on inserts. To isolate the paracrine component while enabling pharmacological intervention on the KCs side, conditioned media from treated FBs was applied to KCs. As RNA and protein extraction from cells on transwell inserts is not technically feasible, direct gene knockdown in KCs was used for molecular analyses.

#### Gene knockdown

Gene knockdown was performed using small interfering RNA (siRNA) to downregulate *LIF* (sc-37222, Santa Cruz Biotechnology, USA) or *DSG1* (sc-35224, Santa Cruz Biotechnology, USA). As a control, we used Stealth RNAi siRNA Negative Control Lo GC (12935200, Invitrogen, USA). Cells were transfected using Lipofectamine RNAiMax (13778150, Invitrogen, USA). Knockdown efficiency was confirmed by qRT-PCR, ELISA, and Western blot (Figure S6).

#### Single-cell RNA sequencing and analysis

Skin samples obtained with 4 mm punch biopsies were disassociated using the gentleMACS Dissociator and the Human Whole Skin Dissociation Kit (130-101-540, Miltenyi BioTec, USA) following the manufacturer’s instructions with incubation of 3 h. The dissociated cell suspension was strained through a 40-mM filter and cell viability was measured using Cellometer ViaStain AOPI Staining solution (CS2-0106-5 ML, Revvity, USA). Cells from each sample were loaded into a Chromium Next GEM Chip G (PN-1000127, 10× Genomics, USA) according to manufacturer’s user guide (CG000315, Rev E), aiming for recovery of approximately 10,000 cells per sample. The loaded chip was then placed in a Chromium Controller platform (10× Genomics, USA) to create Gel Beads-in-emulsion (GEMs). Subsequent steps of library preparation were performed using Chromium Next GEM Single Cell 3′ Kit v3.1 (PN-1000269, 10× Genomics, USA). ScRNAseq libraries were sequenced by pair-ended sequencing using NovaSeq 6000 platform (Illumina, USA), aiming for 40,000 reads per cell. ScRNAseq data analysis was processed using a standard computational procedure, using Cell Ranger V6.0 software^68^ to map reads and to quantify gene expression. Cell Ranger filtered count matrices were used for downstream analysis. Different cell type markers genes for each cluster were identified using differentially expressed genes, using Seurat v4.1.1 FindMarkers.^31^

#### Integrated UMAPs

For integration between samples, cells with over 10% of their transcripts originating from mitochondrial genes were excluded from further analysis to filter dead or dying cells. The data was integrated using Seurat v4.1.1 integration pipeline as further described. Datasets were normalized using LogNoramalize. FindVariableFeatures was used for finding highly variable genes (HVGs) for the following stages. Batch correction with CCA and MNN algorithms^31^ was used before integrating the datasets with IntegrateData. The integrated data was then scaled and PCA dimensionality reduction was calculated based on HVGs (npcs = 30). RunUMAP (dim = 1:30), FindNeighbors (dim = 1:30) and FindClusters (resolution = 0.5) were then used for clustering and visualizing the integrated data. The integrated datasets were then visualized using UMAP (using R’s DimPlot).

#### Immunofluorescence

Skin samples obtained with 4 mm punch biopsies were formalin fixed and paraffin-embedded. 5 μm sections were deparaffinized using xylene/ethanol. Antigen retrieval was performed using 0.01 M citrate buffer, pH 6.0 (005000, Invitrogen, USA) in a microwave for 25 min. Cultured KCs were grown on glass coverslips and fixed with 4% formaldehyde. Samples were blocked in 5% bovine serum albumin (BSA) (A7906, Sigma-Aldrich, USA) for 1 h and incubated overnight at 4°C with primary antibodies. Secondary antibody staining was carried out for 1 h at 37°C. Omitting the primary antibody was used for negative control. Imaging was performed using SP8 inverted confocal microscope (Leica Microsystems, Germany) equipped with a Leica HC PL APO CS2 × 63/1.4 NA or Leica HC PL APO CS2 × 20/0.75 NA objectives. Image analysis was performed with Imaris 10 software (BitPlane, USA). For quantification of immunofluorescence, total fluorescence intensity of the channel of interest was measured and divided by the number of DAPI-positive cells within the same region to obtain mean fluorescence intensity per cell.

#### qRT-PCR

RNA was extracted from cultured cells using an RNA extraction kit (11828665001, Roche, Germany). cDNA was synthesized from 1000 ng of total RNA using qScript kit (95047–100, Quanta Biosciences, USA). cDNA PCR amplification was carried out with the PerfeCTa SYBR Green FastMix (95072–012, Quanta Biosciences, USA) on a StepOnePlus system (Applied Biosystems, USA). Each sample was analyzed in triplicates using gene-specific intron-crossing oligonucleotides.

#### ELISA

Cell culture supernatants were collected, aliquoted, and stored at −80°C until analysis. Peripheral blood samples were obtained from patients, centrifuged and stored at −80°C until analysis. All measurements were performed in duplicates. Protein levels of secreted LIF and IL-6 were quantified using commercial ELISA kits (LIF: ab242228, Abcam, UK; IL-6: D6050B, R&D Systems, USA) according to the manufacturers’ protocols.

#### Western blotting

Cells were homogenized in CelLytic M (C2978, Sigma-Aldrich, USA) and a protease inhibitor cocktail (P8340, Sigma-Aldrich, USA). Following centrifugation at 10,000 g for 10 min at 4°C, proteins were electrophoresed through a Gradient Bio-Rad gel (5678093, Bio-Rad, USA) and transferred onto nitrocellulose membrane (1704159, Bio-Rad, USA). After blocking for 1 h using 1 × Tris-buffered saline with Tween 20 (50 mM Tris, 150 mM NaCl, 0.01% Tween 20) with 5% BSA (A7906, Sigma-Aldrich, USA), blots were incubated overnight at 4°C with a primary antibody. The blots were washed five times for 5 min each with 1 × Tris-buffered saline, 0.1% Tween 20 with 1.5% BSA. After incubation with a secondary antibody, and subsequent washings (five times, 5 min each with 1 × Tris-buffered saline Tween 20), proteins were detected using the clarity Western ECL substrate (1705061, Bio-Rad, USA). Protein levels were quantified by ImageJ^67^ software (National Institutes of Health, USA). Uncropped western blot images for all immunoblotting experiments are provided in Methods S1.

#### Dispase dissociation assay

Primary KCs were cultured on 6-well plates until 90% confluence, then exposed to 1.6 μM Ca^2+^ concentration and treatment or control were applied. After 48 h, the cells were incubated in dispase II (04942078001, Roche, Germany) at 37°C for 40 min and detached from the plate as monolayers. Cell sheets were subjected to mechanical stress using inversions. The number of fragments was counted by two independent evaluators. LIF neutralization was performed by using human LIF antibody (0.1 μg/mL, AB-250-NA, R&D systems, USA). IL-6 neutralization was performed by using anti IL-6 antibody (ab6672, Abcam, UK). IL-6 receptor blocking was performed by using tocilizumab (10 μg/mL, Roche, Germany). Neutralizing and blocking antibodies were compared to normal IgG control (AB-108-C, R&D systems, USA). ERK1/2 inhibition was performed using SCH7 72984 (10 μM/mL, Selleckchem, USA); dimethyl sulfoxide (DMSO) was used as vehicle control.

### Quantification and statistical analysis

All *in vitro* experiments were performed with a minimum of three independent biological replicates unless otherwise stated in the figure legends. Data are presented as mean ± SEM. Individual data points are shown in all graphs. Statistical analyses were performed using R. For comparisons between two groups, a Student’s *t* test or paired *t* test was used as appropriate. For comparisons among three or more groups, one-way ANOVA with post-hoc Tukey’s HSD test was applied. scRNAseq differential expression analysis was performed using the Wilcoxon rank-sum test as implemented in Seurat FindMarkers, with Bonferroni-adjusted *p* values reported. PERMANOVA was performed with 999 permutations using Euclidean distance. A *p* value <0.05 was considered statistically significant. Specific statistical tests, sample sizes, and *p* values for each experiment are reported in the corresponding figure legends. Asterisks in figures denote: ∗*p* < 0.05, ∗∗*p* < 0.001, ∗∗∗*p* < 0.0001.
