# Supplementary material for: Interleukin 4–driven loss of stromal LIF signaling affects immune responses and cell-cell adhesion in atopic dermatitis
Source: iScience. 2026 Jul 13;29(8):116766. doi: 10.1016/j.isci.2026.116766 (PMC13382125; doi:10.1016/j.isci.2026.116766)
Supplement: Document S1. Figures S1–S6, Tables S1 and S2, and Methods S1 [file mmc1.pdf]

iScience, Volume *29*

Supplemental information

Interleukin 4–driven loss of stromal LIF signaling affects immune responses and cell-cell adhesion in atopic dermatitis

Yarden Feller, Kiril Malovitski, Sari Assaf, Yan Stein, Sivan Friedman, Dafna Tussia-Cohen, Lubna Khair, Rawaa Ishtewy, Tzachi Hagai, Avraham Unterman, Ofer Sarig, Eli Sprecher, and Liat Samuelov

Supplementary Figures S1-S6, Tables S1-S2, Methods S1

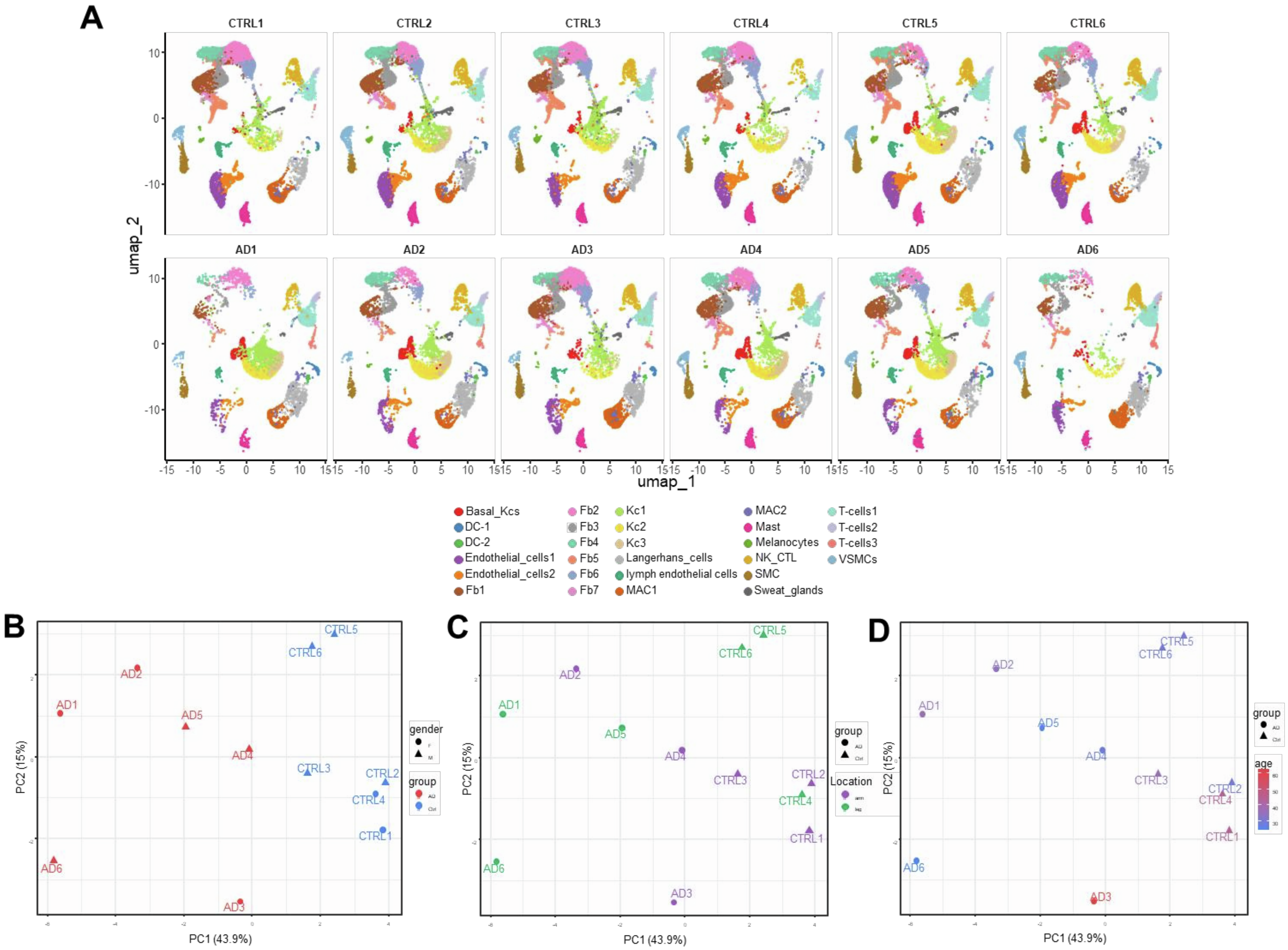
A
CTRL1
CTRL2
CTRL3
CTRL4
CTRL5
CTRL6
AD1
AD2
AD3
AD4
AD5
AD6
umap_2
umap_1
Basal_Kcs
DC-1
DC-2
Endothelial_cells1
Endothelial_cells2
Fb1
Fb2
Fb3
Fb4
Fb5
Fb6
Fb7
Kc1
Kc2
Kc3
Langerhans_cells
lymph endothelial cells
MAC1
MAC2
Mast
Melanocytes
NK_CTL
SMC
Sweat_glands
T-cells1
T-cells2
T-cells3
VSMCs
B
C
D
PC1 (43.9%)
PC2 (15%)
gender
group
Location
age

Figure S1. Inter-individual variability analysis of donors.

(A) Uniform Manifold Approximation and Projection (UMAP) projections of all 108,145 cells split by individual donor, colored by cell type. Upper row: six healthy control donors (CTRL1–CTRL6); lower row: six AD donors (AD1–AD6). (Atopic dermatitis (AD), Keratinocytes (Kcs), Dendritic cells (DC), Fibroblasts (Fb), Macrophages (MAC), Natural Killer cells (NK), Cytotoxic T-cells (CTL), Smooth muscle cells (SMC), Vascular smooth muscle cells (VSMCs)); Principal component analysis (PCA) of donor cell-type composition profiles. Each point represents one donor (n = 12). **(B)** Donors colored by disease status (AD, red; healthy, blue) and shaped by sex (circle, female; triangle, male); **(C)** donors colored by sampling location (arm, purple; leg, green); **(D)** donors colored by age (blue, younger; red, older).

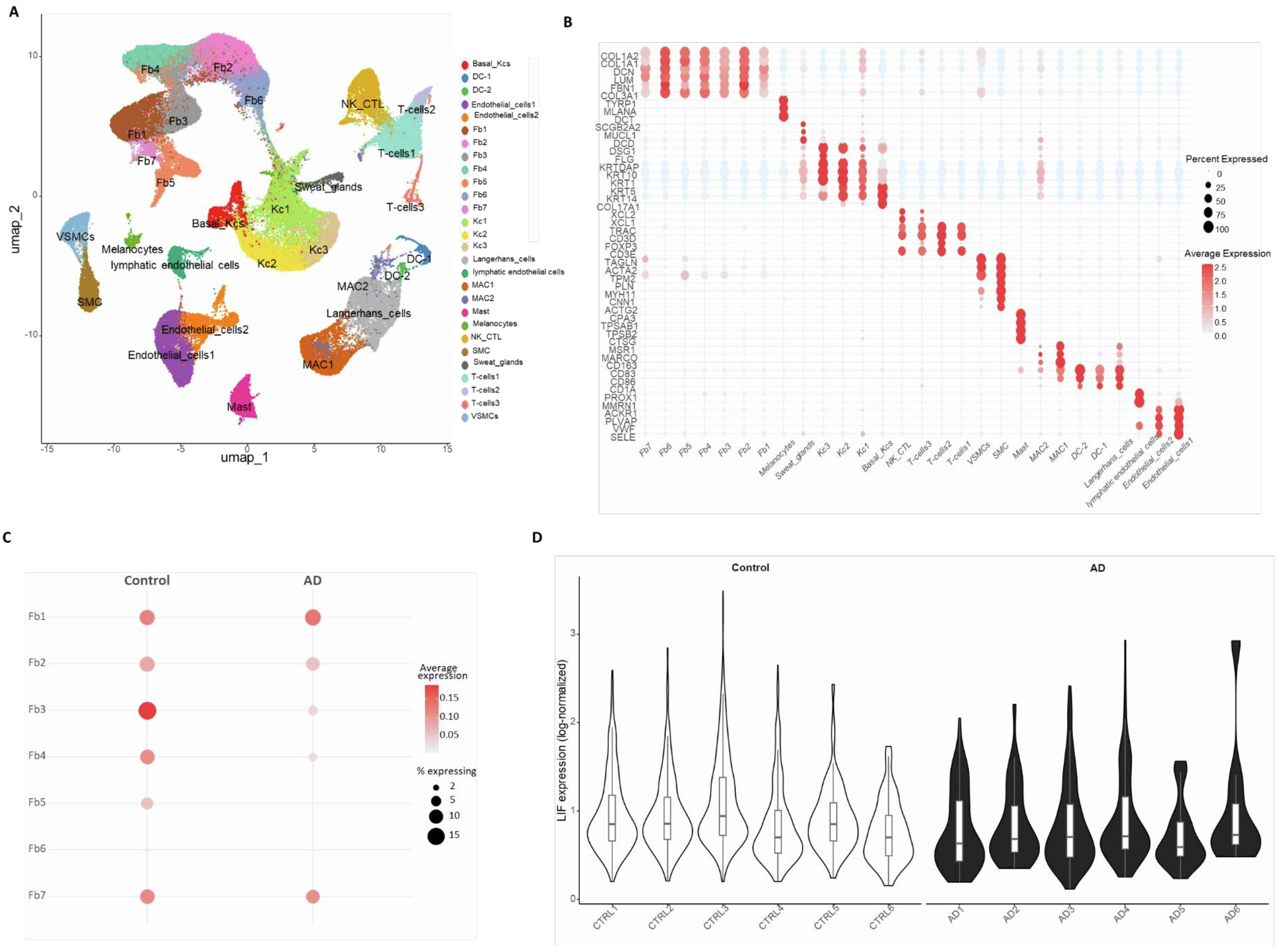
A
B
C
D
umap_1
umap_2
Basal_Kcs
DC-1
DC-2
Endothelial_cells1
Endothelial_cells2
Fb1
Fb2
Fb3
Fb4
Fb5
Fb6
Fb7
Kc1
Kc2
Kc3
Langerhans_cells
lymphatic endothelial cells
MAC1
MAC2
Mast
Melanocytes
NK_CTL
SMC
Sweat_glands
T-cells1
T-cells2
T-cells3
VSMCs
COL1A2
COL1A1
DCN
LUM
FBN1
COL3A1
TYRP1
MLANA
DCT
SCGB2A2
MUCL1
DCD
DSG1
FLG
KRTDAP
KRT10
KRT1
KRT5
KRT14
COL17A1
XCL2
XCL1
TRAC
CD3D
FOXP3
CD3E
TAGLN
ACTA2
TPM2
PLN
MYH11
CNN1
ACTG2
CPA3
TPSAB1
TPSB2
CTSG
MSR1
MARCO
CD163
CD83
CD86
CD1A
PROX1
MMRN1
ACKR1
PLVAP
VWF
SELE
Percent Expressed
Average Expression
Control
AD
Average expression
% expressing
LIF expression (log-normalized)
CTRL1
CTRL2
CTRL3
CTRL4
CTRL5
CTRL6
AD1
AD2
AD3
AD4
AD5
AD6

Figure S2. Single-cell characterization of dermal fibroblast populations and LIF expression in AD skin.

(A) UMAP projection of 108,145 high-quality cells from lesional skin of 6 AD patients and 6 healthy controls, colored by cell type. (Atopic dermatitis (AD), Keratinocytes (Kcs), Dendritic cells (DC), Fibroblasts (Fb), Macrophages (MAC), Natural Killer cells (NK), Cytotoxic T-cells (CTL), Smooth muscle cells (SMC), Vascular smooth muscle cells (VSMCs)); **(B)** dot plot showing expression of marker genes used to annotate the 28 cell populations. Dot size represents the percentage of cells expressing each marker; dot color represents average expression level; **(C)** dot plot showing *LIF* expression across the 7 fibroblast subpopulations in AD patients and healthy controls. Dot size represents the percentage of LIF-expressing cells; dot color represents average LIF expression; **(D)** violin plots showing *LIF* expression among LIF-expressing fibroblasts from each individual donor, split by disease group (AD, blue; controls, white). Each violin represents one donor. Expression levels are shown for LIF-expressing cells only.

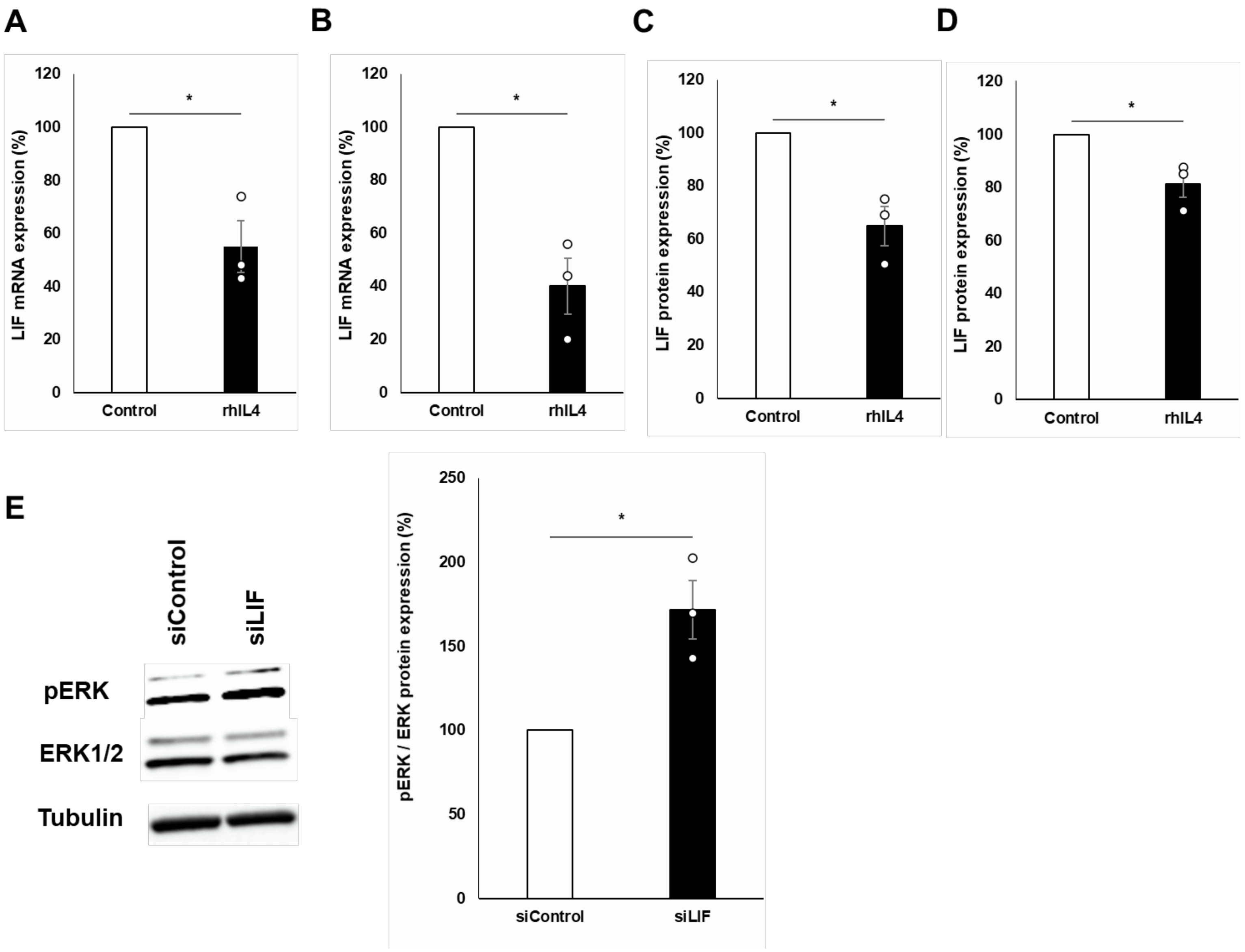
A
B
C
D
E
LIF mRNA expression (%)
LIF protein expression (%)
Control
rhIL4
*
siControl
siLIF
pERK
ERK1/2
Tubulin
pERK / ERK protein expression (%)

Figure S3. Time-dependent effect of IL-4 on LIF expression and pERK activation.

(A–B) Fibroblasts (FBs) were supplemented with 20 ng/ml rhIL-4 and cultured for 24 and 48 hours. Total RNA was extracted and *LIF* mRNA levels were quantified by qRTPCR, normalized to *ACTB*. Individual data points represent values from each independent experiment. Data are shown as mean ± SEM of three independent experiments, expressed as percentage relative to control; paired t-test; **(C–D)** FBs were supplemented with 20 ng/ml rhIL-4 and cultured for 24 and 48 hours. LIF protein levels in supernatants were quantified by ELISA. Individual data points represent values from each independent experiment. Data are shown as mean ± SEM of three independent experiments, expressed as percentage relative to control; paired t-test; **(E)** KCs were transfected with siLIF or siControl. ERK1/2 and pERK expression were assessed by immunoblotting. α-tubulin immunoblotting served as a loading control. Positions of molecular weight markers (kDa) are indicated on the left. Individual data points represent values from each independent experiment. Data are shown as mean ± SEM of three independent experiments, expressed as percentage relative to siControl; t-test. Asterisks denote: $^*P < 0.05$.

A

B

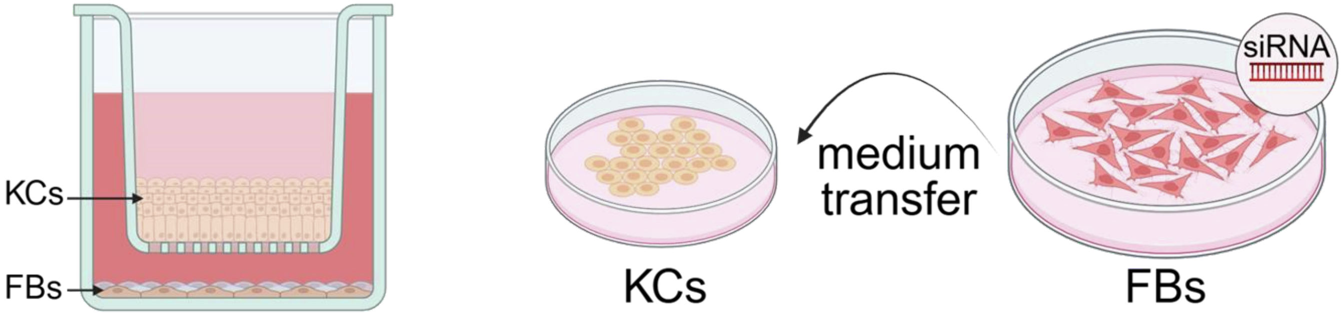

Figure S4. Graphical illustration of co-culture experiments.

(A) Fibroblasts (FBs) were cultured in a 6-well plate and treated with siRNA. Keratinocytes (KCs) were cultured in a transwell mesh insert above the FBs; **(B)** FBs were cultured in a 6-well plate and treated with siRNA. After 24 hours, conditioned medium was transferred to KCs cultured in a separate well. Created with BioRender.com.

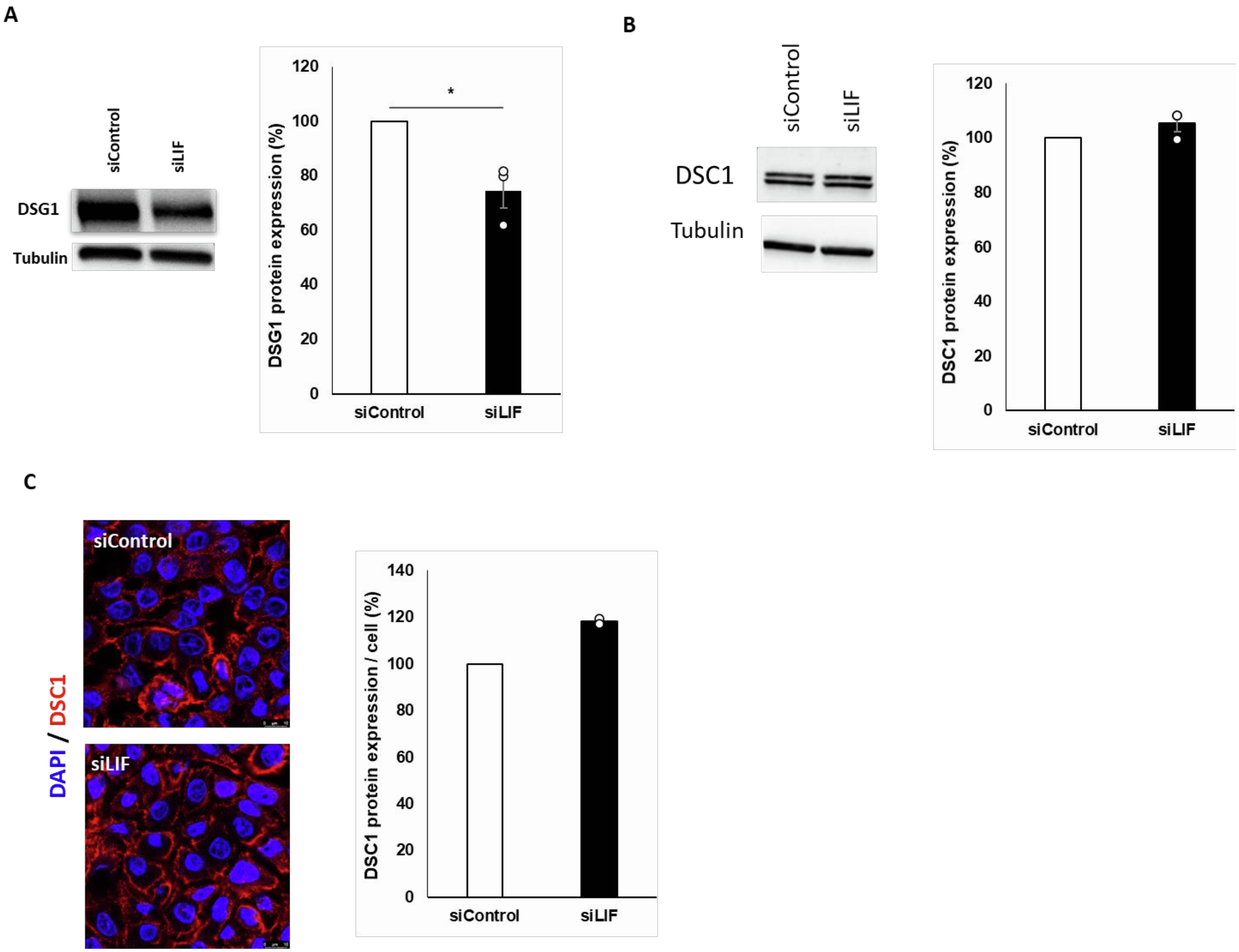
A
siControl
siLIF
DSG1
Tubulin
DSG1 protein expression (%)
120
100
80
60
40
20
0
*
siControl
siLIF
B
siControl
siLIF
DSC1
Tubulin
DSC1 protein expression (%)
120
100
80
60
40
20
0
siControl
siLIF
C
siControl
siLIF
DAPI / DSC1
DSC1 protein expression / cell (%)
140
120
100
80
60
40
20
0
siControl
siLIF

Figure S5. Expression of DSG1 and DSC1 following LIF knockdown.

(A) KCs were transfected with si*LIF* or siControl. DSG1 expression was assessed by immunoblotting. α-tubulin immunoblotting served as a loading control. Positions of molecular weight markers (kDa) are indicated on the left. Individual data points represent values from each independent experiment. Data are shown as mean ± SEM of three independent experiments, expressed as percentage relative to siControl; t-test; **(B)** KCs were transfected with si*LIF* or siControl. Desmocollin 1 (DSC1) expression was assessed by immunoblotting. α-tubulin immunoblotting served as a loading control. Positions of molecular weight markers (kDa) are indicated on the left. Individual data points represent values from each independent experiment. Data are shown as mean ± SEM of three independent experiments, expressed as percentage relative to siControl; **(C)** KCs were transfected with si*LIF* or siControl, then stained for DSC1 (red) and DAPI (blue) (scale bar = 10 μm). Quantification represents mean fluorescence intensity normalized to the number of $DAPI^+$ cells. Individual data points represent values from each independent experiment. Data are shown as mean ± SEM of two independent experiments, expressed as percentage relative to siControl. Asterisks denote: *$P < 0.05$.

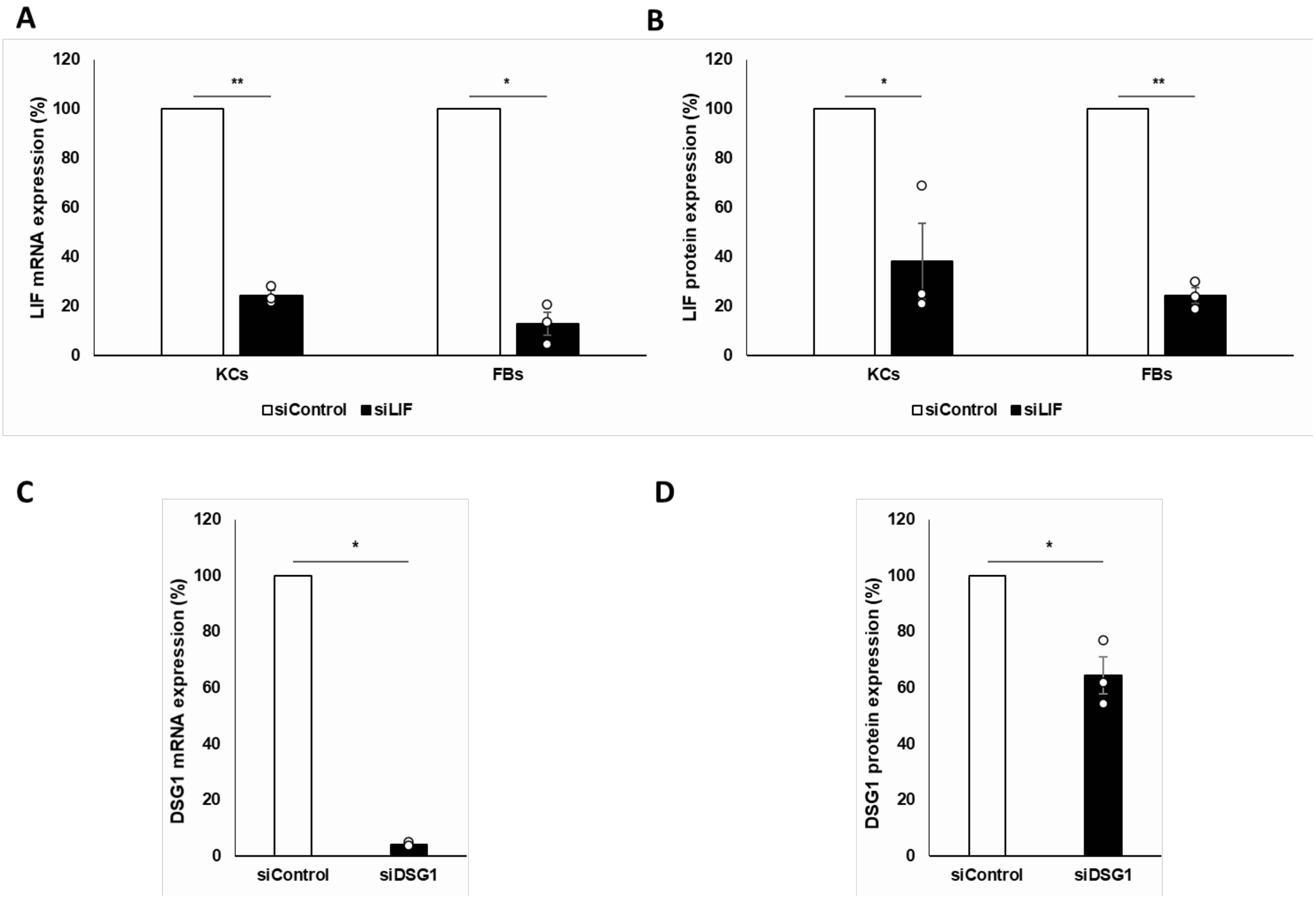
A
LIF mRNA expression (%)
120
100
80
60
40
20
0
**
*
KCs
FBs
siControl
siLIF
B
LIF protein expression (%)
120
100
80
60
40
20
0
*
**
KCs
FBs
siControl
siLIF
C
DSG1 mRNA expression (%)
120
100
80
60
40
20
0
*
siControl
siDSG1
D
DSG1 protein expression (%)
120
100
80
60
40
20
0
*
siControl
siDSG1

Figure S6. Validation of siRNA activity.

(A) Keratinocytes (KCs) and fibroblasts (FBs) were transfected with si*LIF* or siControl and cultured for 48 hours. Total RNA was extracted and *LIF* mRNA levels were quantified by qRT-PCR, normalized to *ACTB*. Individual data points represent values from each independent experiment. Data are shown as mean ± SEM of three independent experiments, expressed as percentage relative to control; paired t-test; **(B)** LIF protein levels in supernatants were quantified by ELISA. Individual data points represent values from each independent experiment. Data are shown as mean ± SEM of three independent experiments, expressed as percentage relative to siControl; paired t-test; **(C)** KCs were transfected with si*DSG1* or siControl. Total RNA was extracted and *DSG1* mRNA levels were quantified by qRT-PCR, normalized to *GAPDH*. Individual data points represent values from each independent experiment. Data are shown as mean ± SEM of three independent experiments, expressed as percentage relative to siControl; paired t-test; **(D)** DSG1 protein expression was assessed by immunoblotting. α-tubulin immunoblotting served as a loading control. Positions of molecular weight markers (kDa) are indicated on the left. Individual data points represent values from each independent experiment. Data are shown as mean ± SEM of three independent experiments, expressed as percentage relative to siControl; paired t-test. Asterisks denote: *P < 0.05, **P < 0.001.

TABLES

Table S1. Skin samples patients' characteristics.

<table>
<tr><th colspan="2" rowspan="2">Characteristic</th><th colspan="2">scRNA-seq</th><th colspan="4">Immunofluorescence</th></tr>
<tr><th>AD (n=6)</th><th>Control (n=6)</th><th>AD (n=6)</th><th colspan="3">Control (n=6)</th></tr>
<tr><td colspan="2">Age, years (mean ± SD)</td><td>34.8 ± 12.9</td><td>35 ± 6.5</td><td>34.8 ± 12.9</td><td colspan="3">43.7 ± 14.8</td></tr>
<tr><td colspan="2">Female, n (%)</td><td>3 (50%)</td><td>2 (33%)</td><td>3 (50%)</td><td colspan="3">3 (50%)</td></tr>
<tr><th colspan="8">Per donor data</th></tr>
<tr><th>Group</th><th>#</th><th>Age (y)</th><th>Sex</th><th>Site</th><th>IGA</th><th>SCORAD</th><th>NRS</th></tr>
<tr><th rowspan="6">AD</th><td>1</td><td>36</td><td>F</td><td>Leg</td><td>3</td><td>39</td><td>1</td></tr>
<tr><td>2</td><td>34</td><td>F</td><td>Arm</td><td>3</td><td>28</td><td>9</td></tr>
<tr><td>3</td><td>62</td><td>F</td><td>Arm</td><td>3</td><td>30</td><td>7</td></tr>
<tr><td>4</td><td>28</td><td>M</td><td>Arm</td><td>3</td><td>31</td><td>3</td></tr>
<tr><td>5</td><td>25</td><td>M</td><td>Leg</td><td>3</td><td>47</td><td>3</td></tr>
<tr><td>6</td><td>24</td><td>M</td><td>Leg</td><td>3</td><td>62</td><td>10</td></tr>
<tr><th rowspan="6">scRNAseq controls</th><td>1</td><td>43</td><td>F</td><td>Arm</td><td></td><td></td><td></td></tr>
<tr><td>2</td><td>29</td><td>M</td><td>Arm</td><td></td><td></td><td></td></tr>
<tr><td>3</td><td>36</td><td>M</td><td>Arm</td><td></td><td></td><td></td></tr>
<tr><td>4</td><td>44</td><td>F</td><td>Leg</td><td></td><td></td><td></td></tr>
<tr><td>5</td><td>29</td><td>M</td><td>Leg</td><td></td><td></td><td></td></tr>
<tr><td>6</td><td>29</td><td>M</td><td>Leg</td><td></td><td></td><td></td></tr>
<tr><th rowspan="6">Immunofluorescence controls</th><td>1</td><td>58</td><td>M</td><td>Leg</td><td></td><td></td><td></td></tr>
<tr><td>2</td><td>19</td><td>M</td><td>Arm</td><td></td><td></td><td></td></tr>
<tr><td>3</td><td>29</td><td>M</td><td>Leg</td><td></td><td></td><td></td></tr>
<tr><td>4</td><td>59</td><td>F</td><td>Arm</td><td></td><td></td><td></td></tr>
<tr><td>5</td><td>49</td><td>F</td><td>Arm</td><td></td><td></td><td></td></tr>
<tr><td>6</td><td>48</td><td>F</td><td>Arm</td><td></td><td></td><td></td></tr>
</table>

Table S2. PERMANOVA table

PERMANOVA results quantifying the contribution of each covariate to donor cell-type compositional variance (Euclidean distance, 999 permutations).

Covariate	R^2	F_stat	p_value
Disease (AD vs Healthy)	19.2%	2.38	0.042
Gender	8.6%	0.94	0.432
Age group	19%	1.06	0.383
Sampling location	10.6%	1.19	0.314

Methods S1. Uncropped western blot images of all immunoblotting experiments performed in this study.

Figure S2d:

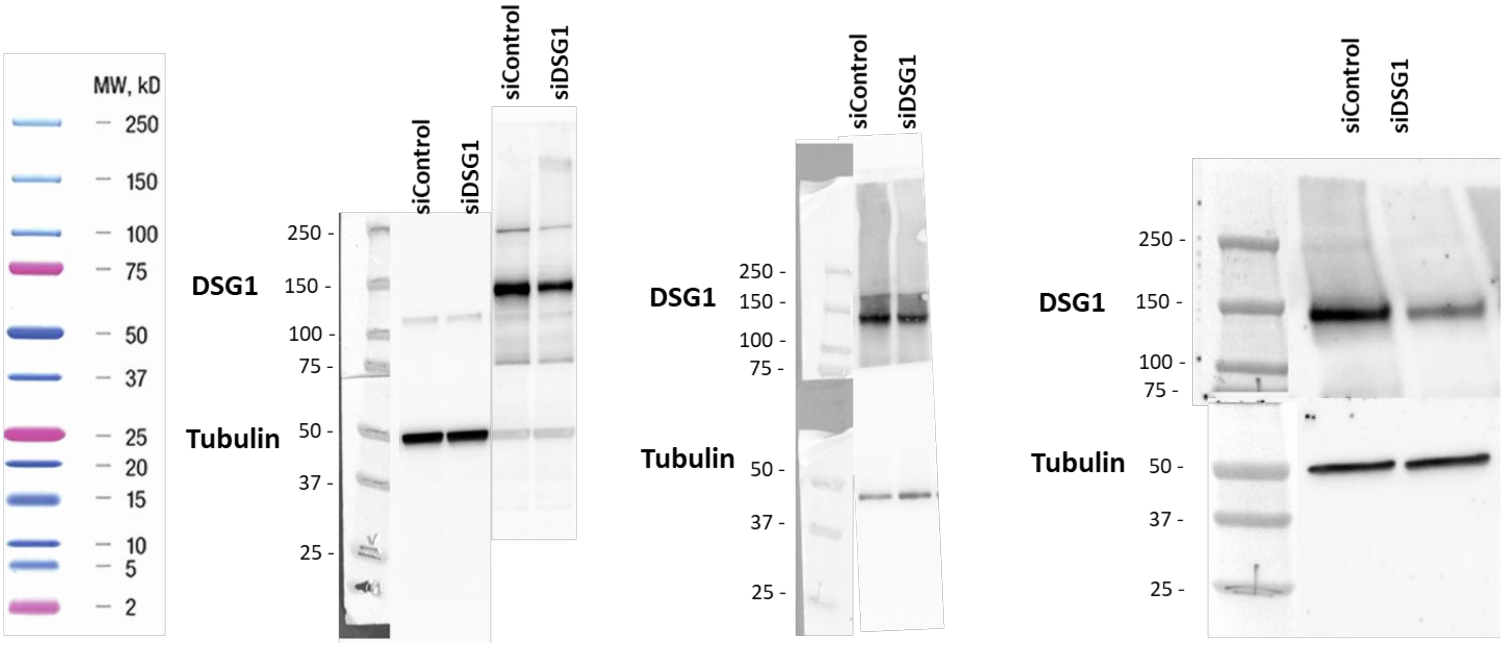

Figure S4e:

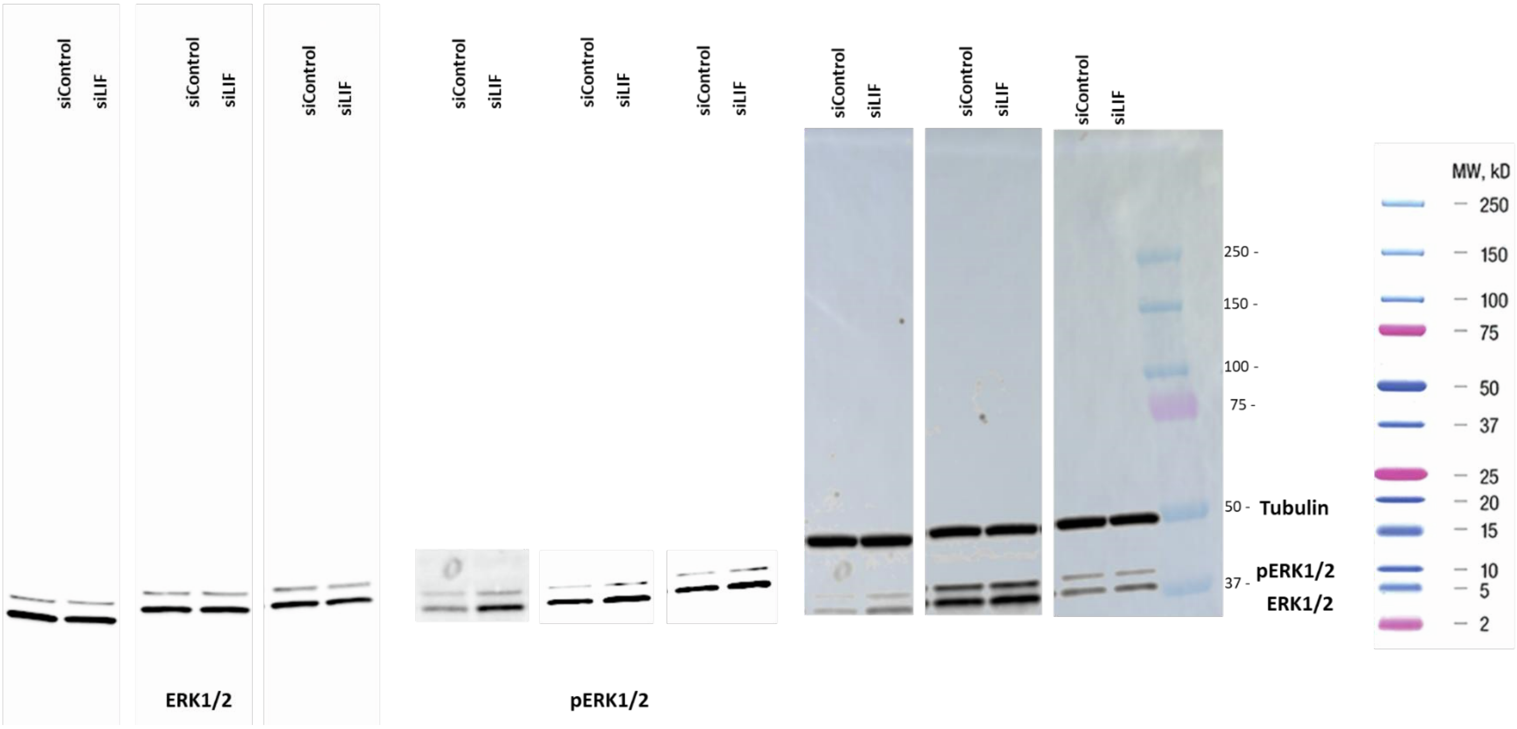

Figure S6a:

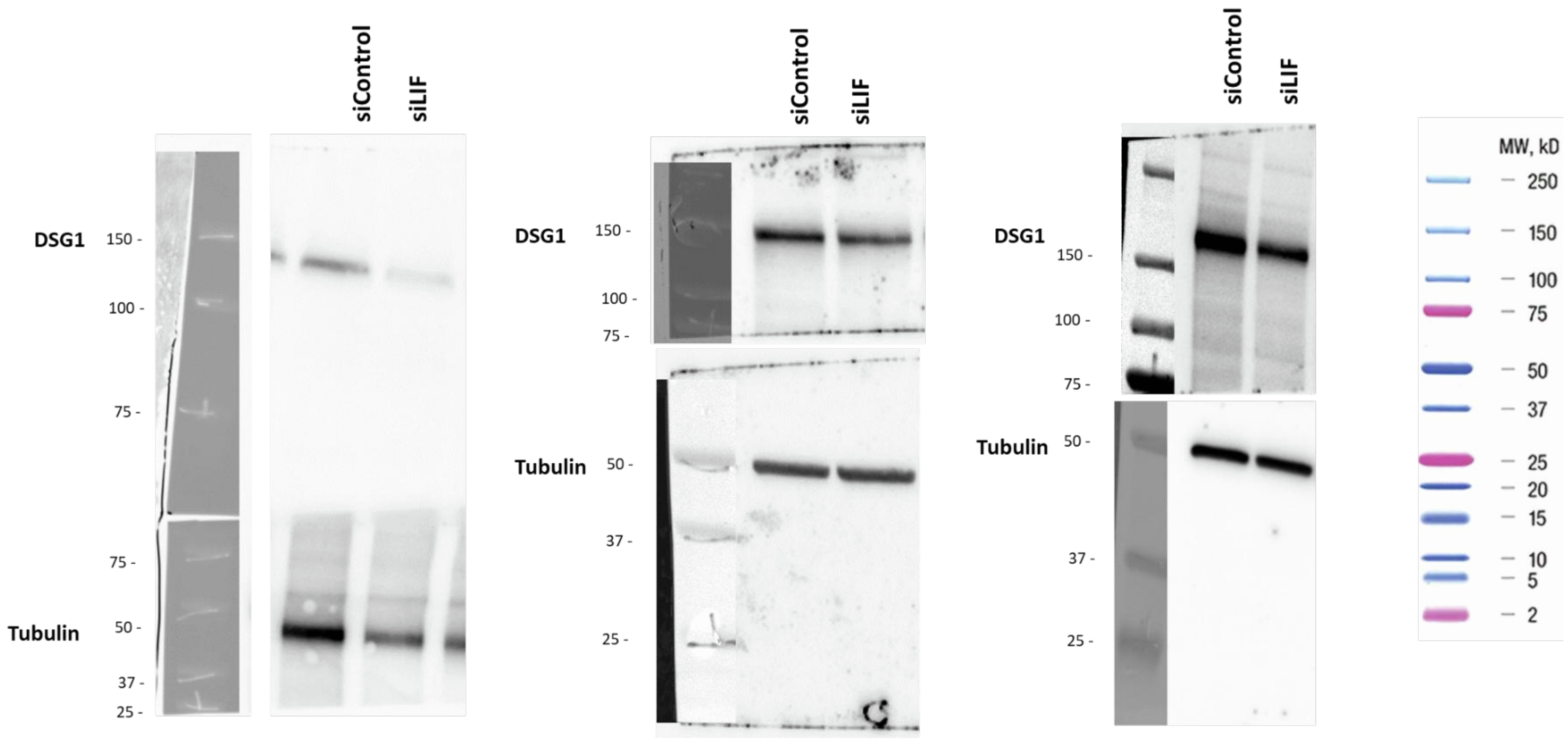
siControl
siLIF
DSG1
Tubulin
150 -
100 -
75 -
75 -
50 -
37 -
25 -
siControl
siLIF
DSG1
150 -
100 -
75 -
Tubulin
50 -
37 -
25 -
siControl
siLIF
DSG1
150 -
100 -
75 -
Tubulin
50 -
37 -
25 -
MW, kD
250
150
100
75
50
37
25
20
15
10
5
2

Figure S6b:

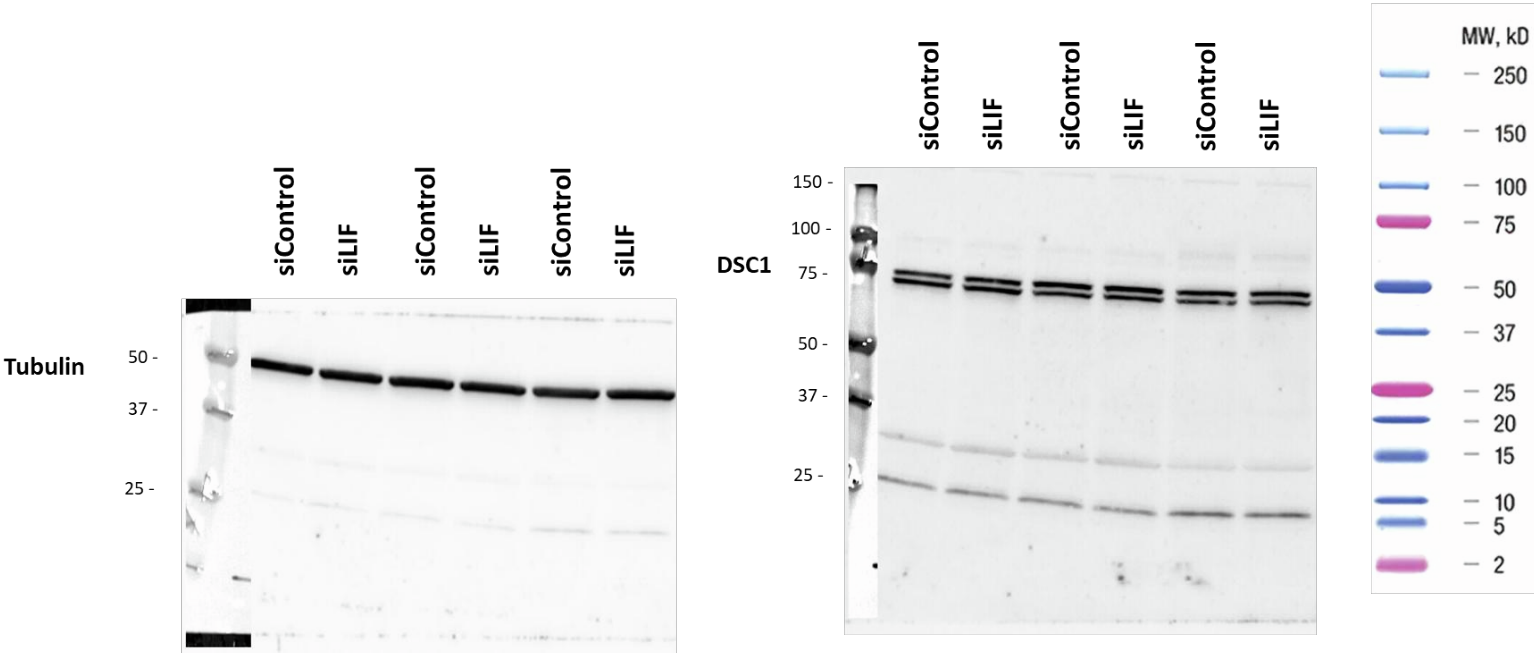
siControl
siLIF
siControl
siLIF
siControl
siLIF
Tubulin
50 -
37 -
25 -
siControl
siLIF
siControl
siLIF
siControl
siLIF
DSC1
150 -
100 -
75 -
50 -
37 -
25 -
MW, kD
250
150
100
75
50
37
25
20
15
10
5
2
